# A Comprehensive Review on Schisandrin B and Its Biological Properties

**DOI:** 10.1155/2020/2172740

**Published:** 2020-03-14

**Authors:** M. I. Nasser, Shuoji Zhu, Chen Chen, Mingyi Zhao, Huanlei Huang, Ping Zhu

**Affiliations:** Guangdong Cardiovascular Institute, Guangdong Provincial People's Hospital, Guangdong Academy of Medical Sciences, Guangzhou, Guangdong 510100, China

## Abstract

Nature is a vast source of bioactive molecules and has provided an active and efficient reservoir for drug discovery. Among natural compounds, one of the most promising is Schisandrin B (Sch B), isolated from Schisandra chinensis, which was documented to possess diversified pharmacokinetic propriety, among them antioxidant, anti-inflammation, cardioprotection, and neuroprotection. Due to its large biological properties, Sch B was recorded to be a potent cure for several diseases by targeting several signaling pathways. This review is aimed at emphasizing the recent data on the biological properties of Sch B among the molecular mechanism of this drug on tumoral, cardiac, and neural diseases. The data suggest that the antitumor activities of Sch B were mainly through apoptosis and cell cycle arrest at the diver's stage. It is reported that Sch B could be used as effective chemotherapy, neuroprotection, and cardioprotection since it possesses a spectrum of biological activities; however, further investigations on the mechanism of its action and preclinical trials are still mandatory to further validate the potential of this natural drug candidate.

## 1. Introduction

Natural compounds have been broadly used since ancient times to prevent and cure various illnesses in Asian countries mostly. Nature attracts particular attention because it provides a vast source of bioactive molecules. Those molecules have been used separately or as a mixture to cure or prevent cancer, cardiovascular disease, and neurodegenerative disease, among others [1, 2]. Among natural compounds, Traditional Chinese Medicine (TCM) has the characteristics of low toxicity, multiple targets, and integrity, which can be regulated by the machinery of the body immune function, inhibit the formation of tumor tissue neovascularization, promote the death of tumor cells, and reduce the effect of tumor cell resistance to achieve antitumor properties, in the prevention and treatment of cancer prescription surface which have certain advantages [3–5]. Among TCM, *Schisandra chinensis* has been used for thousands of years to prevent memory deficiency [6]. With the development of technology, *Schisandra chinensis* is a mixture of several bioactive compounds; among others, we have Sch A, B, and C, which have been used to prevent several illnesses [7, 8]. In the three isoforms of Sch, Sch B attracts particular attention. Previous reports revealed Sch B function in neuroprotection by reducing oxidative stress [9, 10]. In this review, we describe the pivotal role of Sch B in treating cancers, cardiovascular diseases, and neurodegenerative diseases and explain the molecular mechanism as well as the function in several illnesses.

## 2. Schisandrin B and Antioxidation

Modern pharmacological studies have shown that Sch B could play an essential role in liver protection, antioxidation, antiaging, antitumor, antianxiety, and other aspects. Numerous experiments have shown that Sch B could increase the level of superoxide dismutase (SOD) in cells, inhibit lipid peroxidation, and reduce lactate dehydrogenase and malondialdehyde releasing reactive oxygen species therefore directly removing free radicals and playing an antioxidant role [11, 12]. Indeed, the scavenging effect of hydroxyl free radicals of oxygen free radicals by Sch B was most significant, compared to vitamin C at the same concentration [13]. Lam and Ko [10] found that Sch B has specific antioxidant effects on various tissues, including the brain. Earlier studies have shown that Sch B can increase the activity or content of SOD and glutathione (GSH) in tissue cells and resist free radical pairs of biological damage. Recent studies reported that Sch B could also activate glutathione-s-transferase (GST), glutathione reductase (GRD), and glucose-6-phosphate dehydrogenase (G6PD) activity; improve the level of the GSH antioxidant system; and ultimately protect the oxidative body damage [14, 15]. Sch B can also restore the activity of GSH-PX and other antioxidant enzymes and reduce the production of MDA [16, 17].

Moreover, the antioxidative effect of Sch B was also reported on neuroprotective activities. Sch B was found to regulate the expression of the heat shock protein (HSP) gene in neurons [18]. HSP is a component of the subpartner; HSP70 has been proven to have a neuroprotective effect on antioxidant stress, again demonstrating the clinical prevention and treatment of Sch B broad prospects in treating degenerative diseases of the central nervous system. Besides the above pharmacological effects, Sch B can also prevent oxygen-free radicals from forming biofilm structural and functional damage [19, 20]. Likewise, Sch B was reported to avoid ischemia-reperfusion through exerting antioxidant proprieties [21]. Additionally, it is well known that mitochondria are the production sites of ATP and an essential source of reactive oxygen species dysfunction which can produce excessive reactive oxygen species and cause oxidative stress damage, leading to opening of permeability conversion (MPT) holes, and subsequently result in the breakdown of the organelle membrane potential, a process that could trigger ATP synthase to work in reverse, further accelerating ATP depletion, ion homeostasis destruction, and even abnormal cell apoptosis [22]. Furthermore, the moment the MPT hole opens, cytochrome c can leak into the cytoplasm, triggering a series of events such as caspase-9 activation that eventually leads to mitochondria-driven apoptosis [23]. Sch B reduces the production of lipid peroxides, which may be one of its molecular mechanisms to protect mitochondrial integrity [12, 20].

## 3. Schisandrin B and Cancer

Cancer is a pathological condition that has been known since immemorial times by early Egyptians. Despite this ancient lineage (about 3000 B.C.), two modern patterns favor cancers' incidence, and types encountered longevity and lifestyle. For the etymology of cancer, it was derived from the Greek word *karkinos* first coined by Hippocrates (460-370 B.C.) while describing the pathology in its structural aspect as a rounded mass surrounded by radial ramifications like a moving, *clasping crab*. This analogy was an attempt with the breast tumor. He classified cancer based on their size, anatomic sites, and whether it was superficial or deeply embedded at its site [24–26]. Cancer is used as an inclusive name, referring to more than a hundred disease conditions [27]. The pathological conditions are characterized by uncontrolled cell growth beyond boundaries that could start at any anatomical site, then eventually spreading (metastasize) towards the nearby and farthest tissue(s)/organ(s). Like many other epidemic chronic noncommunicable diseases, cancers' incidence is believed to be a consequence of rapid urbanization, environmental pollution, and changes in lifestyles [28, 29].

Chemotherapies remain one of the most used methods for cancer treatment. These drugs inhibited cancer cell growth, therefore stopping the proliferation of cancer [30, 31]. The cytotoxicity of Sch B was reported against a wide variety of human cancer cell lines (Table 1) with low concentration. The cancer cell inhibition by Sch B was demonstrated to be through several biological pathways, among them cell cycle arrest, apoptosis, ROS production, and autophagy.

### 3.1. Sch B Induces Cell Cycle

The cell is the anatomical and physiological unit of life. From that place, cell fulfills all the characteristics of living things, namely, functional organization, metabolism, homeostasis, growth and development, reproduction, passing on genetic information, responding to environmental changes, and ability to adapt through evolution. Cell cycle regulation plays a crucial role in cell death. Every phase of the cell cycle is regulated by the interaction of cyclin and their relevant cyclin-dependent kinases (CDKs), which guaranteed one step to another [32, 33].

**Figure 1:**
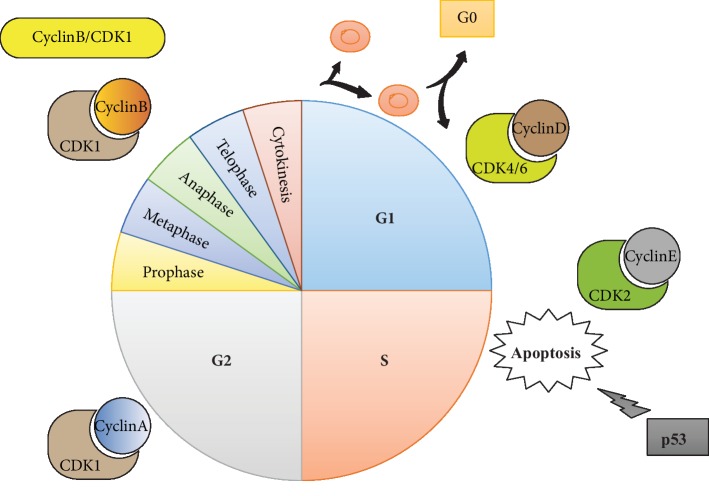
Regulation of cell cycle.

Four stages, including G1, S, G2, and M, are included in the typical cell cycle governed by cyclin-dependent kinases (CDKs) as well as their cyclin partners. Furthermore, the commitment of distribution appears in the G1 stage, which is governed by cyclin D/CDK4/6 as well as cyclin-E/CDK2 at the alleged G1/S changing. In the S stage, DNA is next repeated. Moreover, a second gap stage, namely, the G2 phase, follows this S stage. At the end of G2 stage, entry was governed by cyclin-B/CDK1 into M stage (karyokinesis) at which the cell splits. Under the specific circumstance, they can go into the cell cycle and start splitting again (Figure 1) [34, 35].

Cyclin/cyclin-dependent kinase (CDK) compounds, essential regulators of RNA copying as well as cell cycle advancement, are of great importance. To guarantee suitable progress through every stage, a suite of checkpoints arranged carefully, which regulate diverse cellular kinases needed for unique cell circle events, has been developed by cells. Notably, as for the mitotic entry as well as chromosome segregation, which guarantee the right forming of daughter cells, some cell circle protein kinases which contain members of Polo-like kinases, as well as the Aurora family, are of great significance [36, 37]. Genetic, as well as epigenetic, mechanisms often linked with the proliferation of the tumor cell and the expression of cell cycle managing proteins are generally influenced by these mechanisms, which lead to inadequate checkpoint governance and cause abnormal responses to cellular harm. Both hyperplasic edges and an additional susceptibility to the accumulation of extra genetic changing contributing to the tumor advancement, as well as the gaining of more invasive phenotypes, are caused by these alterations [38, 39]. Researchers have paid particular attention to identifying anticancer medicines directed against crucial cell cycle regulators in the last years. In particular, CDK or cell cycle protein kinase retardants are solved by some preclinical as well as clinical experiments [40, 41].

Sch B was reported to induce inhibition of human lung cancer, cholangiocarcinoma, gallbladder cancer, and gastric cancer cells through which are mediated by inhibition of cyclin D1, as well as CDK4, and CDK6 promoting activation of p21 and p53. Additionally, Sch B was reported to induce lung cancer cell cycle arrest at G2/M phase mainly through the phosphorylation of the checkpoint of the histone H3 at Ser10, which are the monitors of mitosis (chk1). This report also reported that Sch B, by inhibited ATR protein kinase activity, is involved in the G1/S and S phase checkpoint regulation through inhibition of p53 and cdk1 [42]. Previously, we have found that Sch B induces prostate cancer cell (LNCaP) arrest at S phase by inhibition of cyclin E/CDK2, which is associated with an increase of p53 and p21 [43]. Taken together, these data suggest that Sch B might induce cell cycle arrest in all the phases of the cell cycle in the cancer cell and therefore might be used as an efficient drug in the targets of cell cycle arrest to inhibit cancer cell proliferation.

### 3.2. Sch B Induces Cell Apoptosis

Apoptosis is the procedure of programmed cell death (PCD), which might appear in multicellular organisms. Biochemical incidents cause typical cell changes (morphology) as well as death. Chromatin condensation, cell shrinkage, chromosomal DNA fragmentation, and nuclear fragmentation are contained in these variations. Researchers reported that the cell is attracted to commit suicide positively in an advancing and homeostatic circumstance; the incentive of suicide might be offered by the lack of some survival factors; specific change in the morphology occurs in there as well as these suicide paths' biological chemistry; the pathway of “apoptosis” is addressed by someone; and the biochemistry about these suicide paths is followed by a more universal path to delete. However, both of these are generally motivated in a genetic and composite way. There is specific proof that can ensure “apoptosis” symptoms like endonuclease activation. It can be illogically attracted without entering a genetic cascade. Nevertheless, it is necessary to mediate the probably correct apoptosis as well as programmed cell death genetically. In the cancer cell line, apoptosis might occur through a diver's molecular mechanisms [44, 45].

The induction of apoptosis in a cancer cell by Sch B was firstly reported by Wu et al. [46] in human hepatoma SMMC-7721 cells. This was mediated through intrinsic mitochondrial pathways via Hsp70 and caspases-3, 7, and 9, since plentiful studies reported the cytotoxicity-mediated apoptosis in diver's cancer notably through extrinsic pathways. Akt pathway regulates many genes and is implied in cell physiopathology that might contribute to the formation of chemoresistance sensitivity or resistances as well as the formation of solid tumors. Regarding its importance, several studies are nowadays focused on this pathway for further cancer therapy. Sch B was reported to induce cytotoxicity of melanoma, prostate, and glioma cancer cells through inhibition of Akt.

Moreover, it was reported that the Akt activation might be causing phosphorylation of specific proteins that cause cell survival, such as NF-*κ*B, which triggers survival caused by phosphorylation of I*κ*B. The studies of Xiang et al. [47] proved that Sch B induces apoptosis in the gallbladder cell through an apoptosis intrinsic pathway via overexpression of Bax and inhibition of Bcl-2 as well as NF-*κ*B with cleavage of PARP, and caspase3/9.

## 4. Docking System Test

**Figure 2:**
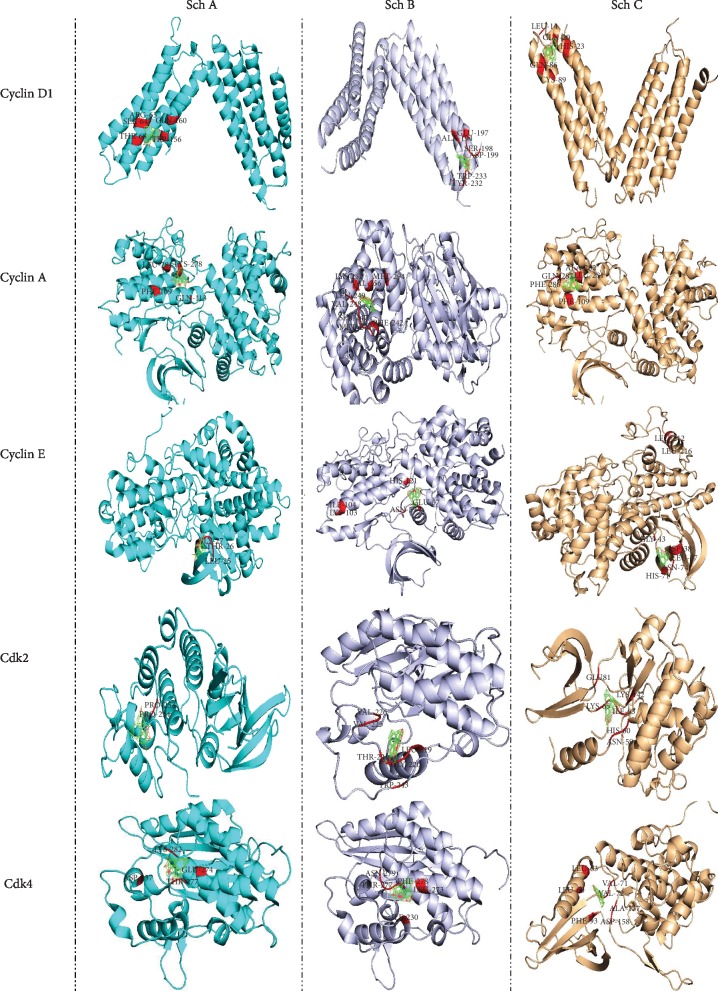
Autodock calculation was performed to determine and compare the binding amino affinity of Sch A, B, and C to cyclins and CDKs that control the cell cycle regulation.

In order to evaluate the efficiency of Sch B compared to Sch A and C, we have performed autodock calculation and then compared the binding affinity as well as binding energies. As observed in Figure 2 and Table 2, Sch B has better binding sites for all cell cycle phases. Indeed, Sch B is able to dock to all cyclin D, E, and A and CDK2 and 4, which are the primary regulator of S, G2, and G1 phases; besides, the binding energy is higher for all cyclin and cyclin-dependent kinase compared to Sch A and C.

**Figure 3:**
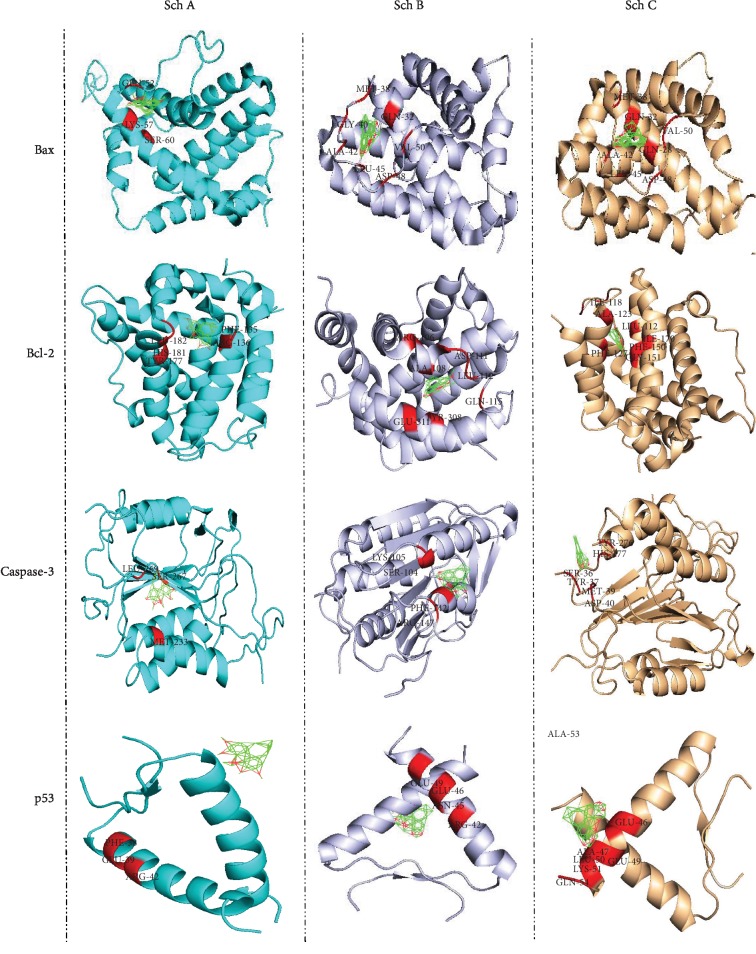
Autodock calculation was performed to determine and compare the binding amino affinity of Sch A, B, and C to p53, Bax, Bcl-2, and caspase-3, which are the principal indicators of apoptosis.

Furthermore, it is well known that p53 acts as a tumor suppressor. P53 plays a crucial role in cell growth arrest or apoptosis depending on the cell type or physiological circumstances. Likewise, p53 also positively regulates the protein expression of Bax while negatively regulating Bcl-2 protein expression. Moreover, a recent study reported p53-null mice to have higher levels of Bax while expressing lower expression of Bcl-2 in many tissues. As observed in Figure 3 and Table 2, Sch B has a better binding affinity to Bcl-2, Bax, p53, and caspase-3 (apoptosomes); furthermore, compared to Sch A and C, it is clear that the binding energy of Sch B to this protein is higher compared to Sch A and C. These data suggest that Sch B could be a better target as chemotherapeutic drugs compared to Sch A and C.

The matrix metalloprotease (MMP) family is a kind of endopeptidase which plays an essential role in the invasion and metastasis of tumor cells. MMPs can promote tumor cells to secrete VEGF, which is secreted by tumor cells promoting the secretion of MMPs by vascular endothelial cells. Among MMPs, MMP-2 and MMP-9 belong to the gelatinases in matrix metalloproteinases, which are closely related to the invasion and metastasis of gastric cancer cells. Furthermore, it was reported that MMP-9 and VEGF could play a crucial role in tumor angiogenesis. Moreover, it was further confirmed that MMP-9, like VEGF, may be associated with the occurrence and development of malignancy and maybe an alternative therapeutic target. As observed in Figure 3(b) and Table 2, Sch B has a better affinity with MMP2/9 as well as VEGF, suggesting that Sch B could be a better target in the treatment of gastric cancer compared to Sch A and C.

**Figure 4:**
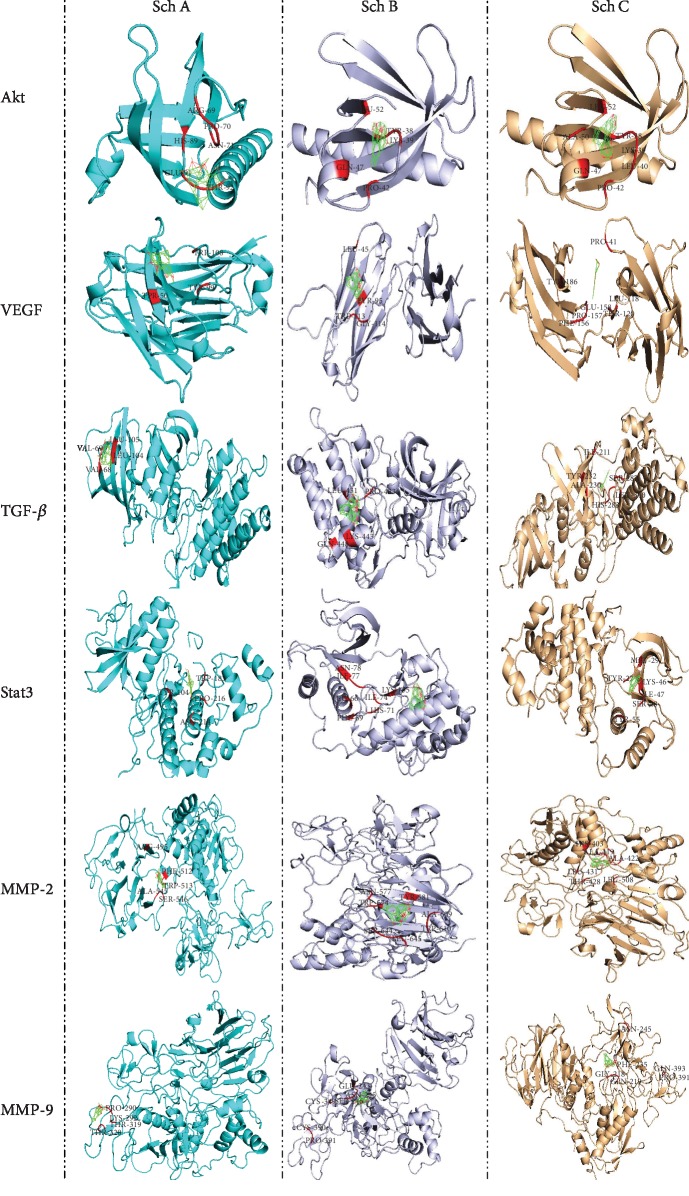
Autodock calculation was performed to determine and compare the binding amino affinity of Sch A, B, and C to the protein that regulated apoptosis pathways.

STAT3 belongs to the family of signal transduction and transcriptional activation. STAT3 plays dual roles in signal transduction and transcriptional activation. The motivated STATs homo- or heterodimerize through mutual SH2 field phosphor-tyrosine roads that are released from the receptor and come into the nucleus, where the transcription of several genetic factors in both rising, as well as adult tissues, is controlled by them. For example, the promoters of Akt, cyclin E, VEGF, Fas, matrix metalloproteinase 2 (MMP2), c-Myc, Mcl-1, HGF, Bcl-xL, and survivin might be bounded to STAT3 in a direct way. As shown in Figure 4 and Table 2, Sch B has a better affinity with STAT3 and Akt compared to Sch A and C. Taken together, Sch B is a better target as chemotherapy drugs for several cancer cells compared to Sch A and C.

## 5. Schisandrin B and Neuroprotection

Neurodegenerative disease (ND) is characterized by the loss of neurons caused by several burdens among Alzheimer's and Parkinson's diseases and cerebrovascular impairment. Although those diseases have a similar symptom, their mechanism differs. Scientists reported that ND is mainly associated with microglia inflammation, and it is the accumulation of those microglia which are responsible for the induction of neurodegenerence through the release of proinflammatory factors such as tumor necrosis factor (TNF), interleukin (IL-6/1*β*), and nitric oxide (NO) [58, 59]. Those are mainly responsible for neurodegenerence via apoptosis depending on the neuroinflammatory process. Therefore, target novel drugs that might inhibit neuroinflammation and prevent neuron death through apoptosis become preferential targets by researchers to palliative neurodegenerence diseases. *S. chinensis* has been used since ancestral time in China as a supertonic for brain disorders and has been proven to reverse ischemia as well as improve cognition. Recent studies revealed that Sch B is the main active compound in the use of *S. chinensis* for neuroprotection. In fact, Sch B was shown to inhibit the protein expression of IL-1*β* and TNF-*α* with phosphorylation of MMP-2/9 in the ischemic hemispheres and, therefore, protect rat cerebrum against inflammation as well as metalloproteinase degradation [60]. On the other hand, Sch B was also shown to avoid microglial-mediated inflammatory through inhibited proinflammatory cytokine such as TNF-*α*, IL-6, PGE2, and NO. The anti-inflammatory activity of Sch B was also related to its ability to interact between Toll-like receptor and their adapter proteins (MyD88, IRAK-1, and TRAF-6), which consequently induce the suppression of IKK transcription factor through NF-*κ*B pathways [61]. Besides, Sch B was shown to reduce oxidative stress by inducing inhibition of MDA and increase SOD expression and therefore prevent hind limb I/R muscle injury [62].

Moreover, recently, several reports have proven that Nrf2 signaling pathways play a role in the antioxidative response element gene by reducing oxidative stress in several diseases, among them cancer, Alzheimer's and Parkinson's diseases, and ischemia. Owning its antioxidant properties, Sch B was reported to protect neurodegenerence through inhibition of ROS and malondialdehyde while increasing glutathione and dismutase levels through Nrf2 pathways [63]. Moreover, in Parkinson's disease, Sch B could decrease cell survival by upregulating the miR34a expression and inhibiting Nrf2 pathways [64]. Finally, in Alzheimer's disease, Sch B was reported to reduce GSK-3*β*, a key enzyme necessary for the hyperphosphorylation of tan protein, therefore contributing to the protection of neurons from Alzheimer's diseases [65].  Table 3 below resumes the recent application of Sch B as neuroprotection.

## 6. Schisandrin B and Myocardial Ischemia

Worldwide, cardiovascular diseases (CVD) remain one of the most leading causes of mortality. Among CVD, myocardial infarction is the main reason for morbidity in developing countries. Till now, the most efficient cure of myocardial infarction consisted of reperfusion therapy; however, reperfusion could induce inflammatory response or oxidative stress, consequently inducing cellular death. This phenomenon was reported as ischemia/reperfusion (I/R) injury. Regarding this, researchers' hypothesis identification of novel pharmaceutical drugs that might exert as well as reduce anti-inflammation could prevent the I/R injury in myocardial injury [66–68].

Some mechanism has been reported to prove the efficiency of Sch B in the treatment of myocardial ischemia. Earliest, heat shock treatment could increase myocardial Hsp25 and Hsp70 expressions that could protect against I-R injury under the present experimental conditions. Sch B was reported to prevent I/R enhancing the expression of Hsp25 and Hsp70 [69, 70]. Recently, Sch B was said to reduce myocardial injury through inhibition of oxidative stress and induction of Akt phosphorylation and prevent apoptosis by decreasing the cleavage of caspase-3 [66, 71]. Besides, the apoptosis inhibition of Sch B was associated with its ability to downregulate some inflammatory cytokine through eNOS signaling pathways [72]. Moreover, the inhibition of ROS-mediated cardioprotective activity of Sch B was particularly associated with the ability of Sch B to increase mitochondrial glutathione, which consequently enhances myocardial ATP and therefore protects I/R injury [73, 74].

**Figure 5:**
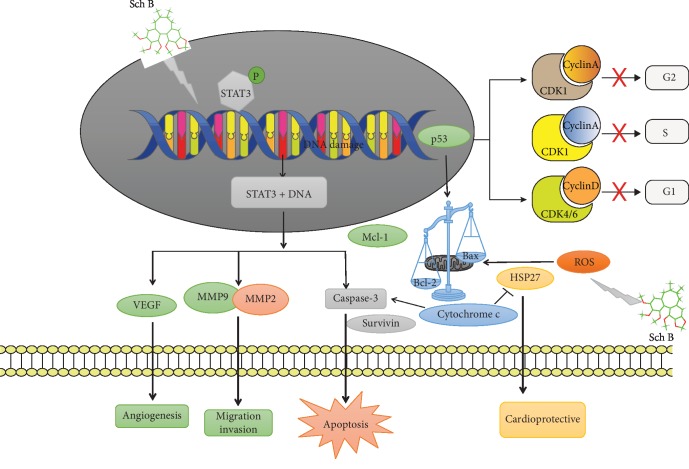
Molecular mechanism of Schisandrin B.

To sum up, the molecular mechanism of cancer cell regulation and cardioprotection by Sch B was resumed in Figure 5.

## 7. Conclusion and Further Perspective

In this review, we have emphasized various pharmacological activities of Sch B. Sch B is a natural nonenzymatic antioxidant with low toxicity and low cost and has a broad application prospect oxidation inhibitor; therefore, it could be used in the cure of many diseases. The antitumor activities of Sch B were mainly associated with the induction of cell cycle arrest at different stages and apoptosis mediated through several signaling pathways. Autodock calculation simulation proved that Sch B is a more efficient drug in the regulation of cell cycle and apoptosis compared to Sch A and Sch C.

Although several molecular and classic pathways have been recognized as possible targets of Sch B in the cancer cell line, the long-term toxicities of Sch B must be evaluated in detail in various animal models to inaugurate its safety profile. Pharmacodynamics biomarkers predictive of Sch B tissue exposure as well as probable response are needed. Moreover, no related research on the clinical application of Sch B has been reported yet. Therefore, preclinical and clinical trials are still required to elucidate the full spectrum of anticancer effects of Sch B, either alone or in synergistic combination with existing therapies.

## Figures and Tables

**Table 1:** Antitumoral and molecular target of Sch B on several cancer cell lines.

Type of cancer	Cell lines	Targets	Effects	References
Colon	CACO2 HCT116	FAK ↓	Antiulcer	[48]
Prostate	DU145 LNCaP	PI3K, AKT, STA3, JAK2, CDK2, cyclin E ↓, p53, p21 ↑	Apoptosis S phase arrest	[43]
Breast	MDA-MB-231, BT-549, MDA-MB-468, MCF-7	STAT3, DOX ↑, Survivin, TGF-*β* ↓	Apoptosis Metastasis Cell cycle arrest S phase ROS production	[49–51]
Ovarian	A2780	DOX ↑ Survivin ↓	Apoptosis	[50]
Melanoma	B16F10	AKT ↓	Hyperpigmentation	[50]
Lung	A549	TGF-*β*1, Bcl-2, HIF-1, VEGF, MMP-9, MMP-2, cyclin D1, CDK6, CDK4 ↓, p53, p21 ↑	Cycle arrest at G2/M checkpoint and G0/G1 Apoptosis	[42, 52, 53]
Glioma	U251, U87	HOTAIR, p-Akt, p-Mtor MMP-9, *ΔΨ*m ↓	Apoptosis	[54–56]
Cholangiocarcinoma		*ΔΨ*m, cyclin D1, Bcl-2, CDK-4 ↓	G0/G1 phase arrest Apoptosis	[57]
Gallbladder	GBC-SD and NOZ	Bax ↑ Bcl-2, NF-*κ*B, cyclin D1 CDK-4 ↓	G0/G1 phase arrest Apoptosis	[47]
Gastric	SCG-7901	Cyclin D1, mRNA ↓	G0/G1 phase arrest	[47]
Hepatoma	SMMC-7721	Hsp70 ↓	Apoptosis	[46]

**Table 2:** Binding acid amine, as well as the binding energy of Sch A, B, and C, was evaluated through autodock calculations.

Protein	Schisandrin		
	A	B	C
Bax	GLN52, LYS57, SER60 (-0.83)	GLN32, MET38, GLY40, ALA42, LEU45, ASP48, VAL50 (-7.0)	GLN28, GLN32, MET38, ALA42, LEU45, ASP48, VAL50, PRO51 (-7.43)
Bcl-2	PHE135, ARG136, TYR177, HIS181, LEU182 (-2.02)	ARG106, ALA108, SER110, ASP111, LEU112, GLN115, TYR 308, GLU311 (-8.1)	LEU112, LEU116, ILE118, ALA123, PHE127, PHE150, GLY151, LEU154, ILE170, TYR308 (-7.92)
P53	PHE38, ARG42, GLU39 (-0.66)	ARG42, ASN45, GLU46, GLU49 (-6.43)	GLU46, ALA47, GLU49, LEU50, LYS51, ALA53, GLN54 (-5.16)
Akt	ARG69, PRO70, ASN71, HIS89 GLU91, THR92 (-1.38)	TYR38, LYS39, GLN47, PRO42, LEU52 (-7.69)	TYR38, LYS39, LEU40, PRO42, GLN47, ALA50 LEU52 (-6.6)
Caspase-3	MET233, SER267, LEU269 (-1.01)	SER104, LYS105, PHE142, ARG147 (-6.42)	SER36, TYR37, MET39, ASP40, TYR274, TYR276, HIS277 (-4.6)
Cdk2	PRO253, PRO254 (-0.29)	GLY220, THR221, LEU219, VAL225, VAL226, TRP243 (-7.16)	ASN59, HIS60, ILE63, LYS65, GLU81, LYS142 (-6.58)
Cdk4	ASP237, GLU274, THR277, LYS282 (-0.59)	ILE230, LEU273, THR277, ASN279, PHE278 (-8.13)	LEU60, LEU63, VAL71, VAL72, PHE93, ALA157, ASP158 (-5.93)
Cyclin D	SER64, ARG65, THR68, TRP156, GLN160 (0.4)	ALA194, GLU197, SER198, ASP199, TYR232, TRP233 (-7.42)	LEU14, HIS23, GLN20, GLN86, CYS89 (-6.65)
Cylin E	LEU25, THR26, GLY27 (-0.25)	GLU57, ASN59, LYS103, ILE104, HIS121 (-7.24)	LEU37, ASP38, GLY43, HIS71, ASN74, LEU212, LEU216 (-5.7)
Cyclin A	PHE109, GLN113, LYS278, LEU281 (-0.06)	PHE242, MET246, SER247, VAL248, LEU249, LYS252, VAL256, MET294 (-6.86)	PHE109, LEU281, ALA282, PHE286, GLN287 (-6.49)
MMP-2	ARG495, PHE512, TRP513, ALA545, SER546 (-0.2)	TRP574, ASN577, TYR581, ALA609, TRP610, SER644, LEU645, GLN643 (-6.67)	HIS403, PRO417, ALA419, LEU420, ALA422, ILE424, THR428, LEU431, LEU508 (-7.03)
MMP-9	PRO290, LYS292, THR319, THR320 (0.01)	GLU243, ASN245, SER246, CYS349, CYS390, PRO391 (-7.43)	GLY218, GLN219, PHE235, ASN245, PRO391, GLN393 (-6.64)
STAT3	TYR104, TRP183, ASN214, PRO216 (-0.84)	LEU66, PHE69, HIS71, ILE74, GLY76, ILE77, ASN78, LYS155 (-6.72)	TYR27, MET29, LYS46, ILE47, SER48, TYR55 (-6.72)
TGF-*β*	VAL68, VAL69, LEU104, LEU105 (-0.14)	LEU431, PRO433, LYS443, GLN448 (-6.39)	ILE211, ALA230, TYR232, HIS283, GLU284, GLY286, SER287, LEU340 (-6.97)
VEGF	TPR50, TYR99, TRP108 (-0.69)	LEU45, TYR95, TRP113, GLY114 (-6.24)	PRO41, LEU118, THR120, PRO157, PHE156, GLU158, TYR186 (-5.52)

**Table 3:** Molecular mechanism of neuroprotection by Sch B.

Assay	Organism tested	Dose/conc.	Mechanisms	References
Sch B prevents neuron from Alzheimer's disease	Mice	340, 290, 80, and 70 nM	Inhibited glycogen synthase kinase-3*β*, therefore alleviating the cell injury induced by A*β* and the cognitive disorders in AD	[65]
Sch B prevents neurodegenerence induced by anxiety associated with oxidative stress	Mice	30 mg/kg and 60 mg/kg	Increase the activity of SOD, GSH, and Nrf2 and Keap1, along with suppression of ROS production	[63]
Sch B might improve Parkinson's disease	Mice	100 *μ*M	Improve 6-OHDA-induced neural cell death and activated Nrf2 signaling pathways along with boosting the expression of miR-34a. Those protect from neurodegenerence	[64]
Sch B prevents cerebral ischemia	Sprague-Dawley rats	10 and 30 mg/kg	Inhibited the expression of proinflammatory factor (TNF-*α* and IL-1*β*) as well as prevented the activation of MMP2/9	[60]
